# A binary matrix factorization algorithm for protein complex prediction

**DOI:** 10.1186/1477-5956-9-S1-S18

**Published:** 2011-10-14

**Authors:** Shikui Tu, Runsheng Chen, Lei Xu

**Affiliations:** 1 Department of Computer Science and Engineering, The Chinese University of Hong Kong, Shatin, N.T., Hong Kong; 2 Bioinformatics Laboratory and National Laboratory of Biomacromolecules, Institute of Biophysics, Chinese Academy of Sciences, Beijing 100101

## Abstract

**Background:**

Identifying biologically relevant protein complexes from a large protein-protein interaction (PPI) network, is essential to understand the organization of biological systems. However, high-throughput experimental techniques that can produce a large amount of PPIs are known to yield non-negligible rates of false-positives and false-negatives, making the protein complexes difficult to be identified.

**Results:**

We propose a binary matrix factorization (BMF) algorithm under the Bayesian Ying-Yang (BYY) harmony learning, to detect protein complexes by clustering the proteins which share similar interactions through factorizing the binary adjacent matrix of a PPI network. The proposed BYY-BMF algorithm automatically determines the cluster number while this number is pre-given for most existing BMF algorithms. Also, BYY-BMF’s clustering results does not depend on any parameters or thresholds, unlike the Markov Cluster Algorithm (MCL) that relies on a so-called inflation parameter. On synthetic PPI networks, the predictions evaluated by the known annotated complexes indicate that BYY-BMF is more robust than MCL for most cases. On real PPI networks from the MIPS and DIP databases, BYY-BMF obtains a better balanced prediction accuracies than MCL and a spectral analysis method, while MCL has its own advantages, e.g., with good separation values.

## Introduction

Protein-protein interactions (PPI) play key roles in the biological processes including cell cycle control, differentiation, protein folding, signaling, transcription, translation and transport etc. Protein complexes are groups of proteins that densely interact with each another [1]. They are key molecular entities that perform cellular functions. Identifying these interacting functional modules is essential to understand the organization of biological systems. A large amount of protein interactions produced by high-throughput experimental techniques enables us to uncover the protein complexes. However, high-throughput methods are known to yield non-negligible rates of false-positives and false-negatives, due to the limitations of the experimental techniques and the dynamic nature of protein interactions. Thus, it is difficult to accurately predict protein complexes from a PPI network.

PPI networks are generally represented as undirected graphs with nodes being proteins and edges being interactions. Various algorithms have been used to detect subgraphs with high internal connectivity [2-4]. One reputed algorithm is Markov Cluster Algorithm (MCL) [5], which simulates flow in a graph, causes flow to spread out within natural clusters and evaporate inbetween different clusters. The value of a so-called inflation parameter strongly influences the clusters and the cluster number. MCL was used to detect protein families [6], and was shown to be remarkably robust against random edge additions and deletions in quantitative evaluations [3,7]. Particularly, “MCL had the best performance on both simulated and real data sets” [7]. In addition, a spectral clustering (SC) method was introduced in [8] for finding functional modules from a PPI network. Clusters are constructed by selecting a proportion of top absolute values of elements of each eigenvector corresponding to large eigenvalues, and controlling the cluster internal connectivity and cluster-size through thresholds.

In this paper, we propose a binary matrix factorization (BMF) algorithm under Bayesian Ying-Yang (BYY) learning [9,10] to predict protein complexes from PPI networks. The BMF models the binary adjacent matrix *X* of the PPI interaction graph as a product of two low-rank matrices *A* and *Y* with binary entries, i.e., *X* ≈ *AY* , where each column of *Y* represents the interaction pattern of the corresponding protein via weighting the columns of *A*. A cluster consists of proteins sharing similar interaction patterns. The roles of *A* and *Y* are exchangeable due to their symmetric positions in *X* ≈ *AY* , and thus BMF gives a biclustering on both the rows and columns of *X*[11].

We propose a BMF learning algorithm, shortly denoted as BYY-BMF, under the BYY best harmony principle [9]. It has the following merits: (1) It automatically determines the cluster number (or equivalently the low-rank) during the learning process, in contrast to most existing BMF algorithms which require a given cluster number; (2) Its clustering result does not depend on any thresholds or parameters, as opposed to MCL [5] which relies on the inflation parameter for the partition boundaries, as well as SC [8] which strongly depends on thresholds to construct clusters through eigen-decomposition. Moreover, BYY-BMF can be applied to biclustering on a rectangular dyadic matrix.

We adopt the strategy in [3] to test the performance of our algorithm. A test interaction graph is constructed from a set of annotated complexes from the MIPS database [12] by linking the proteins in the same complex, and then altered by random edge additions or deletions under various proportions to simulate the false positives and false negatives in PPI data. The predictions are evaluated with annotated complexes by Sensitivity, Positive-predictive value (PPV), Accuracy and Separation [3]. Since MCL was evaluated in [3] to be more robust than other three popular complex-prediction algorithms on the above four criteria, and regarded in [7] as “the leading technique for direct and module-assisted function prediction”, we focus on comparing BYY-BMF with MCL. By selecting the output with the highest harmony measure under repeated random initializations, BYY-BMF’s predictions are more robust against the false positives and false negatives than MCL’s best predictions with the inflation parameter optimally tuned according to the test performance which is impractical because the test performance is evaluated with the true annotated complexes. Moreover, for real PPI networks from MIPS [12] and DIP [13], the BYY-BMF by averaging all repeated evaluation results is better than MCL (with the most frequently used value for the inflation parameter) and SC, in balancing Sensitivity and PPV. In addition, we demonstrate BYY-BMF’s biclustering performance on synthetic gene expression data given in [14].

## Results and discussion

### A novel binary matrix factorization algorithm under Bayesian Ying-Yang learning

A PPI network is usually represented as an undirected graph *G* = (*V*,*E*) [3,4], where a node *v_i_*(*i* = 1,…,*n*) in *V* represents a protein, and an edge *e* = (*v_i_*, *v_j_*) in *E* represents an interaction between the proteins *v_i_* and *v_j_*. The symmetric adjacent matrix is defined as *X* = [*x_ij_*], where *x_ij_* = 1 if there is an interaction between *v_i_* and *v_j_*, otherwise *x_ij_* = 0. Mathematically, protein complexes are defined as sets of nodes with more edges amongst its members than between its members and the rest. Many methods (see e.g., [4]) were used to detect proteins complexes. A reputed one is called the Markov Cluster Algorithm (MCL) [5], which was shown to be very robust [3].

The adjacent matrix *X* is binary, and analysis on binary data has been studied in the literature, e.g., in [15]. There also have been many efforts on discovering latent binary factors from observation data [16-18]. In this paper, we focus *on X* ≈ *AY*, where *X* = [*x_ij_*]*_n×N_*, *x_ij_* ∈ {0,1}, and *A* = [*a_ij_*]*_n×m_*, *Y* = [*y_jt_*] *_m×N_* , *a_ij_*, *y_jt_* ∈ {0,1}. As in [11],*X* ≈ *AY* equivalently performs a biclustering on the rows (features) of *X* by *A* and on the columns (items) of *X* by *Y*, where each feature/item is assigned to one cluster or more. Most existing BMF algorithms are implemented for a given low-rank *m* (or equivalently the cluster number). For the protein-complex prediction problem, *X* is a symmetric binary adjacent matrix of the PPI network with *n* = *N*, and thus we can further constrain *A* = *Y^T^.* In this paper, we propose a novel BMF algorithm under the Bayesian Ying-Yang (BYY) harmony learning [9,10]. The algorithm is denoted as BYY-BMF or shortly BMF when there is no ambiguity from the context. Our BYY-BMF algorithm considers an effective factorization and an automatic determination of the cluster number simultaneously by maximizing a harmony functional (see eq.(4) in the Section “Methods”), while most existing BMF algorithms need a given cluster number. The computational details are referred to the Section “Methods”.

### Experiments

#### On altered graphs by randomly adding and deleting edges

As in [3], we build a *test graph X* from the MIPS complexes [12] by linking the protein nodes in the same complex. Table 1 evaluates the predicted complexes by various algorithms on the test graph. The “algorithm true” uses the MIPS complexes as the predicted complexes. The BYY-BMF algorithm is implemented with random initialization (*m_init_* = 300, *κ* = 1) by 10^3^ independent trials. The **BMF(avg)** averages the results of all trials, while the **BMF(opt)** denotes the trial with the highest value of the harmony measure (by eq.(4) in Section “Methods”). The **MCL(1.8)** means the MCL process with the inflation parameter being 1.8, while **MCL(opt)** denotes the MCL implementation of possible best Accuracy (Acc), with the optimal inflation parameter value 3.4 for the test graph (see Table (2) in [3], where 1.8 is the most frequent value). Noticing that **BMF(opt)** does not rely on while **MCL(opt)** has to rely on the test performance that needs to know the true annotated complexes, practically it is more interesting to compare whether **MCL(1.8)** is improved by **BMF(avg)** and then further improved by **BMF(opt)** with extra computing cost. SC(10%,1%) means the spectral clustering (SC) is implemented with *α_sc_*% = 10% and *β_sc_%* = 1%.

**Table 1:** Evaluations of different clustering algorithms on the test graph *X*_0,0_

*algorithm*	Sn	PPV	Acc	Sep	#C
**true**	**1.0000**	0.7219	0.8497	0.7826	216

**BMF(opt)**	0.9844	**0.8459**	**0.9125**	**0.8652**	179

**BMF(avg)**	0.9764	0.7805	0.8730	0.7861	147

**MCL(1.8)**	0.9920	0.7689	0.8734	0.8474	157

**MCL(opt)**	0.9818	0.7936	0.8827	0.8560	164

**SC**(10%,1%)	0.6788	0.2661	0.4250	0.0238	622

It presents evaluation results of different clustering algorithms on the test graph *X*_0,0_ (#C: number of predicted complexes).

The observations from Table 1 are as follows. (1) The **BMF(avg)** is improved by the **BMF(opt)** via relieving the local optimum problem with a better initialization guided by the harmony measure at the cost of more computation; (2) The values of the inflation parameter influences MCL’s prediction accuracies; (3) The **BMF(opt)** is better than **MCL(1.8),** and also better than **MCL(opt).**

**Figure 1:**
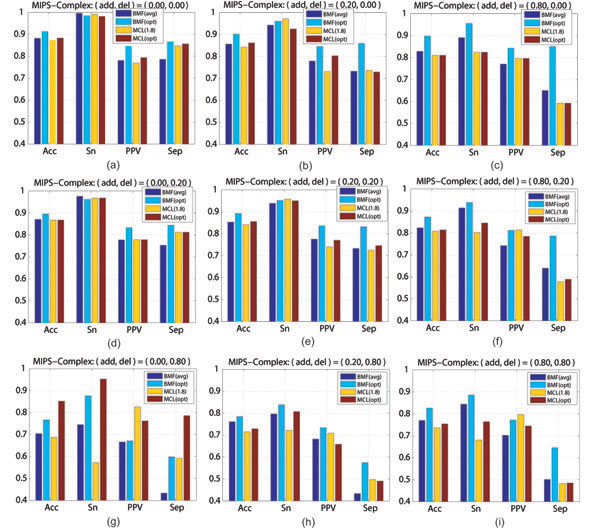
**Prediction evaluations of BMF and MCL on altered graphs** Prediction evaluations of BMF and MCL on altered graphs constructed from a test graph, with *add*% edges randomly added and/or *del*% edges randomly deleted with respect to the original number of edges. To save computation, we actually implement **BMF(opt)** at the same initialization as the one used in Table 1, and use the optimal inflation parameter values given by the Table (2) in [3].

For a systematic evaluation, we alter the test graph *X* to be *X_a_*,*_d_*, where *a* and *d* denote the percentages of randomly added or deleted edges with respect to the number of original edges in *X.* The set of percentage pairs (*a*, *d*) is *P_AD_* = {(*a*, *d*)|*a* ∈ {0, 0.05,0.1, 0.2,0.4, 0.8,1.0}; *d* ∈ {0,0.05, 0.1,0.2, 0.4,0.8} }. A graph *X*_*a*,*d*_ is generated for each of 10 runs of the case (*a*, *d*). The evaluation results, averaged on the 10 runs of each (*a*, *d*) ≠ (0,0), indicate the robustness of each algorithm against false-positive and false-negative edges. To save space, the results on 9 out of 42 percentage pairs (*a*, *d*) in *P_AD_* are presented in Figure 1 (Refer to Additional File 1 for more results).

The value of the prediction Accuracy (Acc) criterion implies how an algorithm balances between Sensitivity (Sn) and PPV. Thus, the “Acc” may serve as a general performance indicator. The observations from Figure 1 are as follows. (1) At the cost of more computation on random repeated initializations, **BMF(opt)** is obviously better than **MCL(1.8).** Moreover, there is still room for improvement via seeking a more effective implementation to replace the current **BMF(opt)** which is based on repeated random initializations. (2) If, on each case (*a*, *d*), allowing to use the information of the true complexes for MCL to tune an optimal inflation parameter value through extra computation of repeatedly trials under different candidate values, **BMF(opt)** is still more robust than **MCL(opt)** for most cases except for (*a*, *d*) = (0, 0.8), a case of a large deletion without any addition. (3) If BYY-BMF is implemented without extra computation as **BMF(avg),** it is still robustly better than or at least comparable to **MCL(1.8)** for majority cases, while **MCL(1.8)** has relative advantages on the Separation (Sep) value for the cases of a large proportion of deletions but a very small percentage of additions.

#### On real PPI data sets

**Figure 2:**
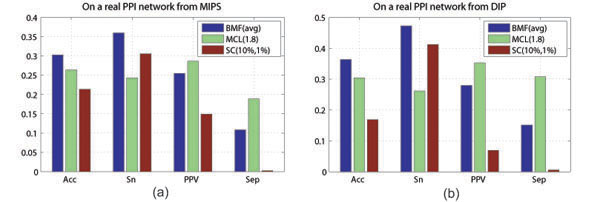
**Prediction accuracies of BMF, MCL and SC on real world PPI networks** Prediction accuracies of BMF, MCL and SC on real world PPI networks are collected from MIPS (*left*) and DIP database (*right*). On a large real PPI network, since it is expensive to implement BMF under repeatedly random initializations and it is not only expensive but also not practical to tune the inflation parameter for MCL using a probably subset of the underlying true complexes as the reference complexes, we implement **BMF(avg)** and **MCL(1.8)**.

Two real PPI data sets are collected from the MIPS [12] and DIP [13]. For a practical comparison and to save computation, we compare BYY-BMF and MCL, by averaging the results of 10 runs of BYY-BMF with *m_init_* = 600, and choosing the most often used inflation parameter value 1.8 for MCL, respectively. We evaluate the predictions with the known 428 reference complexes in Figure 2. The used reference complexes probably cannot cover all true complexes underlying the real PPI networks from MIPS and DIP, and thus as indicated in [3], the values of *PPV* and Separation (Sep) only indicate factional actual complexes annotated already, whereas Sensitivity (Sn) is likely to provide more relevant information of the coverage of the reference complexes recovered in the predictions. The results show that BYY-BMF has a better prediction Accuracy, which balances the Sensitivity and the *PPV*, than MCL, followed by SC, while MCL obtains the best separation value. This observation is consistent with the comparisons between **BMF(avg)** and **MCL(1.8)** from Figure 2 especially for the cases of a small addition proportion but a large deletion proportion. This observation may be reasonable because the real PPI network is very sparse.

#### On gene expression data for biclustering

**Figure 3:**
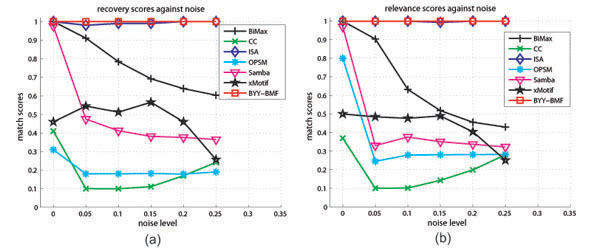
**Matching scores of BYY-BMF versus other biclustering algorithms** The matching score is calculated by DEFINITION 2 in [14]. The bicluster relevance reflects to what extent the generated biclusters represent true biclusters, while the module recovery quantifies how well each true bicluster is recovered. The details of other algorithms are referred to [14].

In addition, we demonstrate to use our BYY-BMF as a biclustering algorithm on synthetic gene expression data in [14]. The original data, which consist of non-overlapping biclusters, are added with random Gaussian noise under increasing noise levels (i.e., the standard deviation). Figure 3 indicates that the performance of BYY-BMF is very robust against noise.

## Conclusions

We have proposed a Binary Matrix Factorization (BMF) algorithm under Bayesian Ying-Yang (BYY) harmony learning, to tackle the problem of predicting protein complexes from a protein-protein interaction (PPI) network. The algorithm has the following merits: (1) The input of the known cluster number required by most existing BMF algorithms is not necessary; (2) As opposed to MCL and SC, BYY-BMF has no dependence on any parameters or thresholds.

Experimental results show that our BYY-BMF algorithm, if implemented by searching the output with the highest BYY harmony measure under repeated random initializations, is more robust against PPI false positives and false negatives than MCL using optimal inflation parameters tuned by the testing accuracies. The prediction results on large real world PPI networks indicate that the average results of repeated independent trials by BYY-BMF obtains a better balanced prediction accuracy, while MCL has a relative advantage in separation value. In addition, we have demonstrated the effectiveness and robustness of BYY-BMF in biclustering on synthetic gene expression data.

Furthermore, the current implementation of BYY-BMF seeks a more optimal performance simply by implementing BYY-BMF at a number of random initializations and selecting one with the highest harmony measure, it suffers high computing costs but indicates that BYY-BMF still has room for improvement via seeking one more effective implementation. Also, BYY-BMF can be extended and used on those data with non-overlapping clusters.

## Methods

### The proposed BYY-BMF algorithm

We present a probabilistic model for the task of binary matrix factorization. The joint likelihood is *q*(*X*, *A*, *Y*, ***θ***) = *q*(*X|A*, *Y*, ***θ***)*q*(*A|****θ***)*q*(*Y|****θ***), where(1)(2)(3)

where both each coloumn of *Y* and each row *A* are contrained to have one and only one “1”. Furthermore, we adopt Dirichlet priors  and  respectively for the parameter ***θ*** = {***α***, ***β***} with hyperparameters , where .

Systematically developed over a decade and half [10], see [9] for a recent overview, the Bayesian Ying-Yang (BYY) harmony learning is a general statistical learning framework for parameter learning and model selection under a best harmony principle. It follows from Eq.(1) and Eq.(2) in [9] that the harmony measure for the above BMF model is the following expression:(4)

where *q*(*·*) gives the Ying representation, and *p*(*·*) gives the Yang representation. All components in Ying representation are given by eq.(1), eq.(2), and eq.(3). In Yang representation, the empirical density *p*(*X*) = *δ*(*X - X_N_*) is adopted with , and the other components are free to be determined via the best harmony, i.e, maximizing *H*(*p||q*).

To achieve the best harmony, a Ying-Yang alternative procedure is implemented and sketched in Algorithm 1. The cluster number starts from a large enough *m_init_* , and reduces during the implementation of this algorithm at its “Model-Selection-Step”. This automatic reduction results from a least complexity nature of maximizing *H*(*p||q*), which can be understood from several perspectives [9]. By one simple interpretation, the maximization forces Ying representation to match Yang representation, but they may not be perfectly equal due to a finite sample size and other constraints. At the equality, *H*(*p||q*) becomes the negative entropy, further maximizing which will minimize system complexity.

This BYY-BMF algorithm reaches an effective factorization and an automatic determination of the cluster number simultaneously, while most existing BMF algorithms need a pre-given cluster number. In the “Yang-Step”, *Y*^(*τ*)^ and *A*^(*τ*)^ are simply computed via individual maximization per column or row. The algorithm results in non-overlapping clusters since there is one and only one “1” per column of *Y* or per row of *A*.

Due to the non-convexity of eq.(4), different initializations BYY-BMF may reach different local optima. To tackle this problem, we implement BYY-BMF at a number of random initializations and select the output with the highest harmony measure. There is a room for more effective implementations.

### Other methods in comparison

MCL [5] simulates flow using two algebraic operations on matrices. The first operation is expansion that models the spreading out of flow, which coincides with normal matrix multiplication. The second is inflation to model the contraction of flow, mathematically a Hadamard power followed by a diagonal scaling. The flow becomes thicker in regions of higher current and thinner in regions of lower current. MCL generates non-overlapping clusters by controlling the flow to spread out within natural clusters and to evaporate inbetween different clusters. The value of an inflation parameter strongly influences the cluster number. The MCL program can be assessed via the web site of Network Analysis Tools (NeAT) [19]. A spectral clustering (SC) method was introduced in [8] to find quasi-cliques (and quasi-bipartites) in a PPI network. First, it calculates the eigen-decomposition *X* = *UDU^T^* for eigenvectors (the columns of *U*) and corresponding eigenvalues (diagonal elements of the diagonal matrix *D*); Then, it constructs clusters by selecting top *α_sc_%* absolute values of each eigenvector corresponding to large eigenvalues; Finally, it discards the nodes linked to less than *β_sc_%* of nodes within a cluster. The obtained clusters depend on the proportion of selection *α_sc_%* and the internal connectivity by *β_sc_%.*

## Data sets

As in [4], the reference protein complexes contain 428 complexes by combining manually curated 216 complexes from MIPS [12], 92 complexes from Aloy et al. [20], and 295 complexes from the SGD database [21]. The PPI network data sets are: (1) constructed from the MIPS complexes by instantiating a node for each protein and linking by an edge any two proteins within the same complex; (2) collected from MIPS database [12], with 12, 317 interactions among 4543 proteins, or from DIP database [13] with 4405 interactions among 2144 proteins. Specifically, the file “Scere20100614CR.txt” from DIP is used.

## Evaluation criteria

To evaluate the accuracy of the predictions, we adopt the following four criteria used in [3,4].

**Sensitivity** (Sn) is defined as follows:(5)

where *n* and *m* is the number of reference and predicted complexes respectively, *T_ij_* is the number of common proteins in the *i*-th reference complex and the *j-th* predicted complex, and *N_i_* is the number of proteins in the *i*-th reference complex. A high *S_n_* value implies a good coverage of proteins in the reference complexes.

**Positive predictive value** (PPV) is defined as(6)

Where . A high PPV value indicates the predicted complexes are likely to be true positive.

**Accuracy** (Acc) is the geometric average of *Sn* and *PPV*,(7)

which balances the complementary information provided by *Sn* and *PPV. Sn* increases to 1 for the big cluster of all proteins, while *PPV* reaches 1 for single-protein clusters.

**Separation** (Sep) value is given by(8)

Where  and . A high *Sep* indicatesa better general correspondence between predicted and reference complexes.

## Competing interests

The authors declare that they have no competing interests.

## Supplementary Material

Additional file 1 In the additional file, all evaluation results on 42 percentage pairs of random additions and deletions are given. Also, a theoretical analysis on the computational efficiency and performance of the proposed BYY-BMF algorithm is presented. Click here for file
