# Supplementary material for: A binary matrix factorization algorithm for protein complex prediction
Source: Proteome Sci. 2011 Oct 14;9(Suppl 1):S18. doi: 10.1186/1477-5956-9-S1-S18 (PMC3724484; doi:10.1186/1477-5956-9-S1-S18)
Supplement: Additional file 1 — In the additional file, all evaluation results on 42 percentage pairs of random additions and deletions are given. Also, a theoretical analysis on the computational efficiency and performance of the proposed BYY-BMF algorithm is presented. [file 1477-5956-9-S1-S18-S1.pdf]

Additional File 1 for “A Binary Matrix Factorization Algorithm for Protein Complex Prediction”

Shikui Tu[1], Runsheng Chen[2,†], and Lei Xu[1,†⋆]

1. Department of Computer Science and Engineering, The Chinese University of Hong Kong, Hong Kong, P.R.CHINA, e-mail: {sktu,lxu}@cse.cuhk.edu.hk.
2. Bioinformatics Laboratory and National Laboratory of Biomacromolecules, Institute of Biophysics, Chinese Academy of Sciences, Beijing 100101
† These authors contributed equally to this work.

Abstract. This file is appended with the paper titled ”*A Binary Matrix Factorization Algorithm for Protein Complex Prediction*” as a supplementary document. In this file, all evaluation results on 42 percentage pairs of random additions and deletions are given. Also, a theoretical analysis on the computational efficiency and performance of the proposed BYY-BMF algorithm is presented. Some parts of theoretical analysis have been included in a working paper titled ”*BYY Harmony Learning Algorithms for Binary Factor Analysis and Binary Matrix Factorization*” to be submitted to the *Neurocomputing* journal.

1 The Evaluations Results of All $6 \times 7 = 42$ Percentage Pairs ($\boldsymbol{add}$,$\boldsymbol{del}$)

As in [1], we build a *test graph* X from the MIPS complexes [2] by linking the protein nodes in the same complex. For a systematic evaluation, we alter the test graph X to be $X_{a,d}$, where a and d denote the percentages of randomly added or deleted edges with respect to the number of original edges in X. The set of percentage pairs (a, d) is $P_{AD} = \{(a, d) \,|\, a \in \{0, 0.05, 0.1, 0.2, 0.4, 0.8, 1.0\};\, d \in \{0, 0.05, 0.1, 0.2, 0.4, 0.8\}\,\}$. We evaluate the predictions on the 42 altered graphs by BYY-BMF(opt) that outputs the clustering result of the highest harmony measure under repeated random initializations, and MCL(opt) that uses the optimal value of the inflation parameter tuned by its prediction performance. All the results are given in Figure 1-10.

2 A Theoretical Analysis on the BYY-BMF algorithm

2.1 Algorithm Details of BYY-BMF

The BYY-BMF algorithm is implemented to maximize the following harmony functional

$$H(p\|q) = \sum_{\boldsymbol{A},Y,X} \int p(\boldsymbol{\alpha}, \boldsymbol{\beta}|X)p(\boldsymbol{A}, Y|X, \boldsymbol{\alpha}, \boldsymbol{\beta})p(X) \ln[q(X|Y, \boldsymbol{A})q(Y|\boldsymbol{\alpha})q(\boldsymbol{A}|\boldsymbol{\beta})q(\boldsymbol{\alpha}|\Xi)q(\boldsymbol{\beta}|\Xi)]d\boldsymbol{\alpha}d\boldsymbol{\beta}. \tag{1}$$

The architecture of the algorithm is sketched in the Section “Methods” of the paper.

In “Yang-Step”, $Y = [\boldsymbol{y}_1, \ldots, \boldsymbol{y}_N]$ is estimated by a discrete optimization, which is simply decoupled into individual maximizations per $\boldsymbol{y}_t$, since the likelihood $q(X|Y, \boldsymbol{A})q(Y|\boldsymbol{\alpha}) = \prod_{t=1}^{N}[q(\boldsymbol{x}_t|\boldsymbol{y}_t, \boldsymbol{A})q(\boldsymbol{y}_t|\boldsymbol{\alpha})]$ is factorizable. It follows that

$$\begin{aligned}\hat{\boldsymbol{y}}_t &= \arg\max_{\boldsymbol{y}_t \in \mathcal{Y}_1} \ln[q(\boldsymbol{x}_t|\boldsymbol{y}_t, \boldsymbol{A})q(\boldsymbol{y}_t|\boldsymbol{\alpha})] \\ &= \arg\max_{\boldsymbol{y}_t \in \mathcal{Y}_1} \left\{ \sum_{i=1}^{n} [x_{it} \ln(1 - e^{-\mathbf{a}_i^T \boldsymbol{y}_t}) - (1 - x_{it})\mathbf{a}_i^T \boldsymbol{y}_t] + \boldsymbol{y}_t^T (\ln \boldsymbol{\alpha}) \right\},\end{aligned} \tag{2}$$

⋆ The correspondence should be addressed to Prof. Lei Xu, lxu@cse.cuhk.edu.hk.

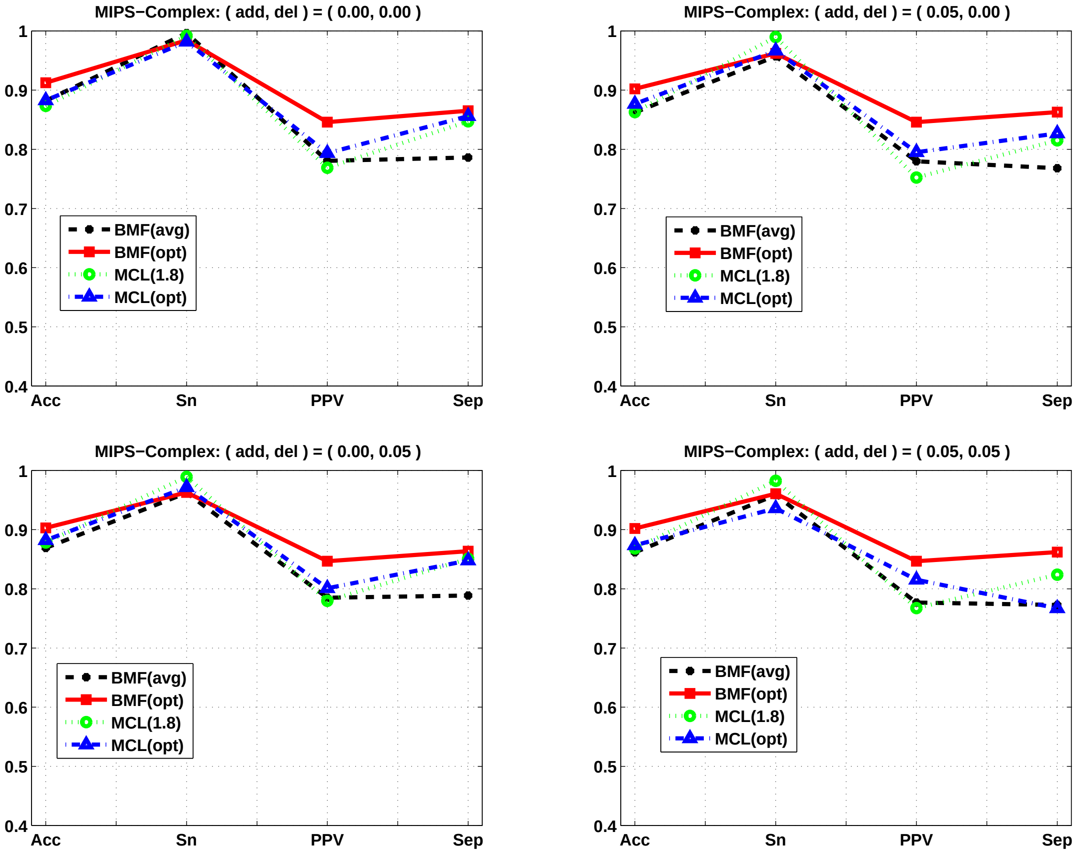

Fig. 1. The evaluations results on 42 percentage pairs (a, d) of random additions and deletions.

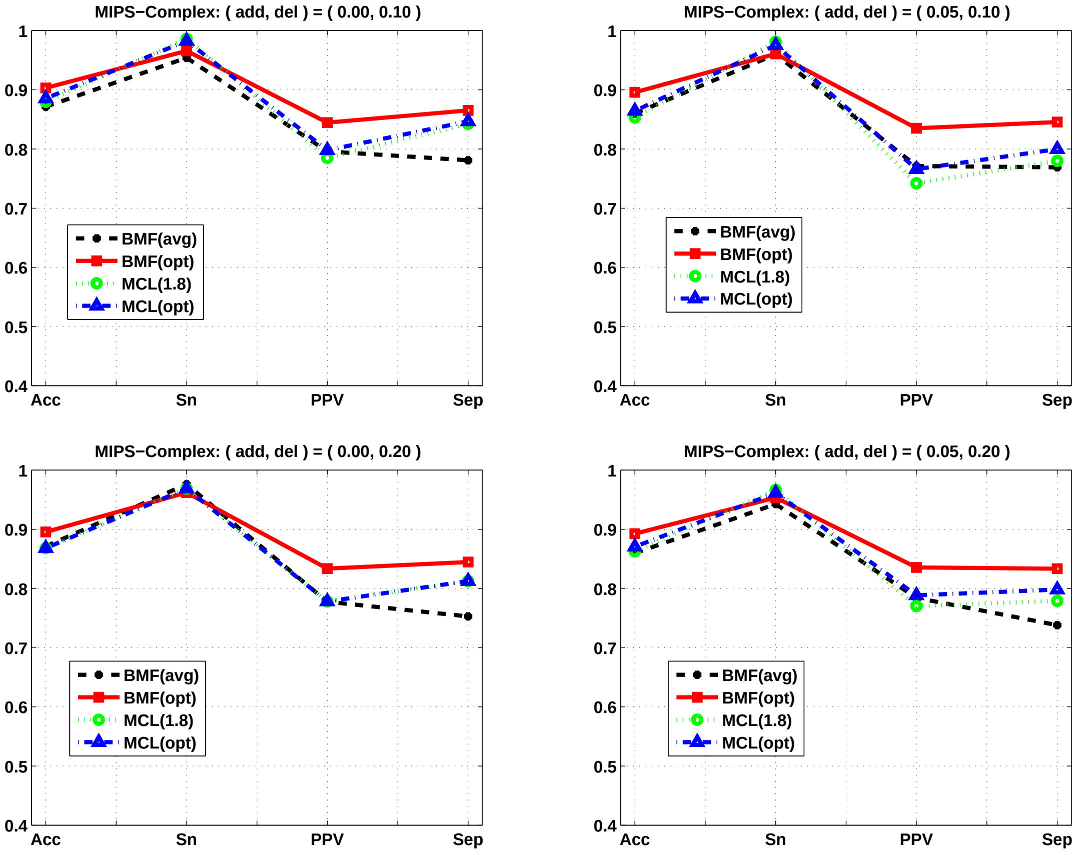

Fig. 2. The evaluations results on 42 percentage pairs (a, d) of random additions and deletions (continue).

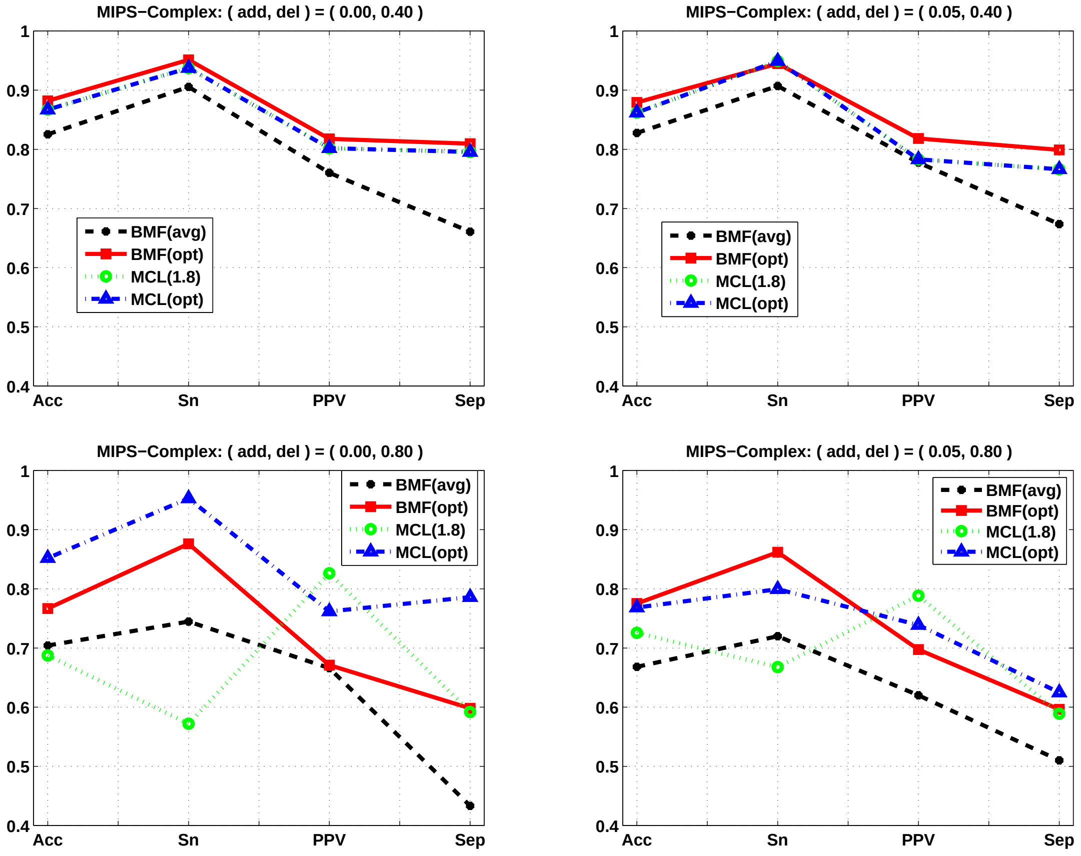

Fig. 3. The evaluations results on 42 percentage pairs (a, d) of random additions and deletions (continue).

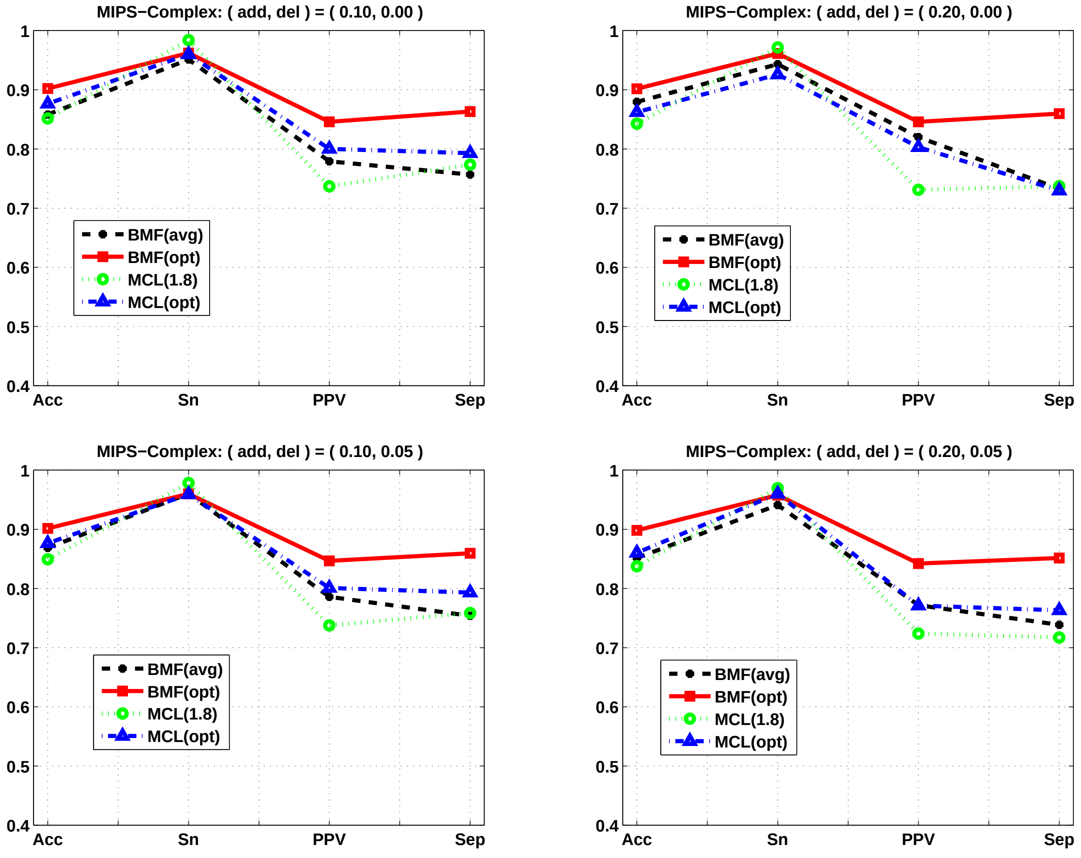

Fig. 4. The evaluations results on 42 percentage pairs (a, d) of random additions and deletions (continue).

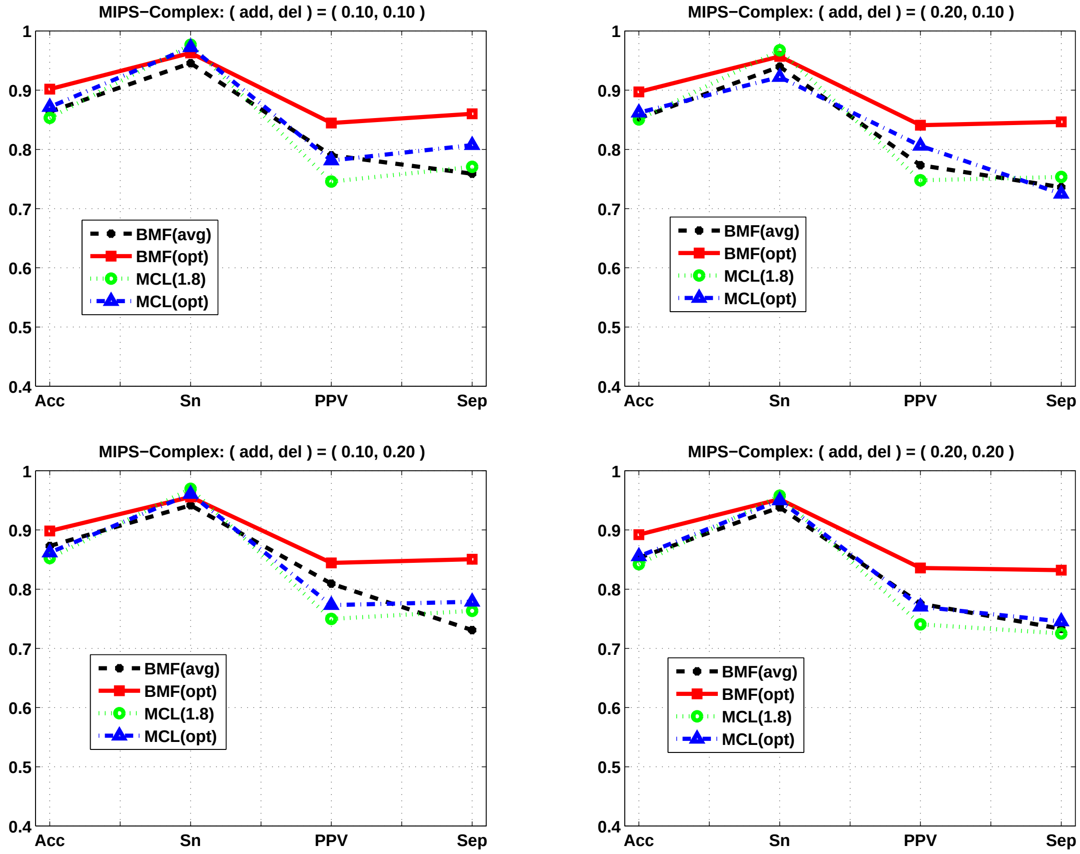

Fig. 5. The evaluations results on 42 percentage pairs (a, d) of random additions and deletions (continue).

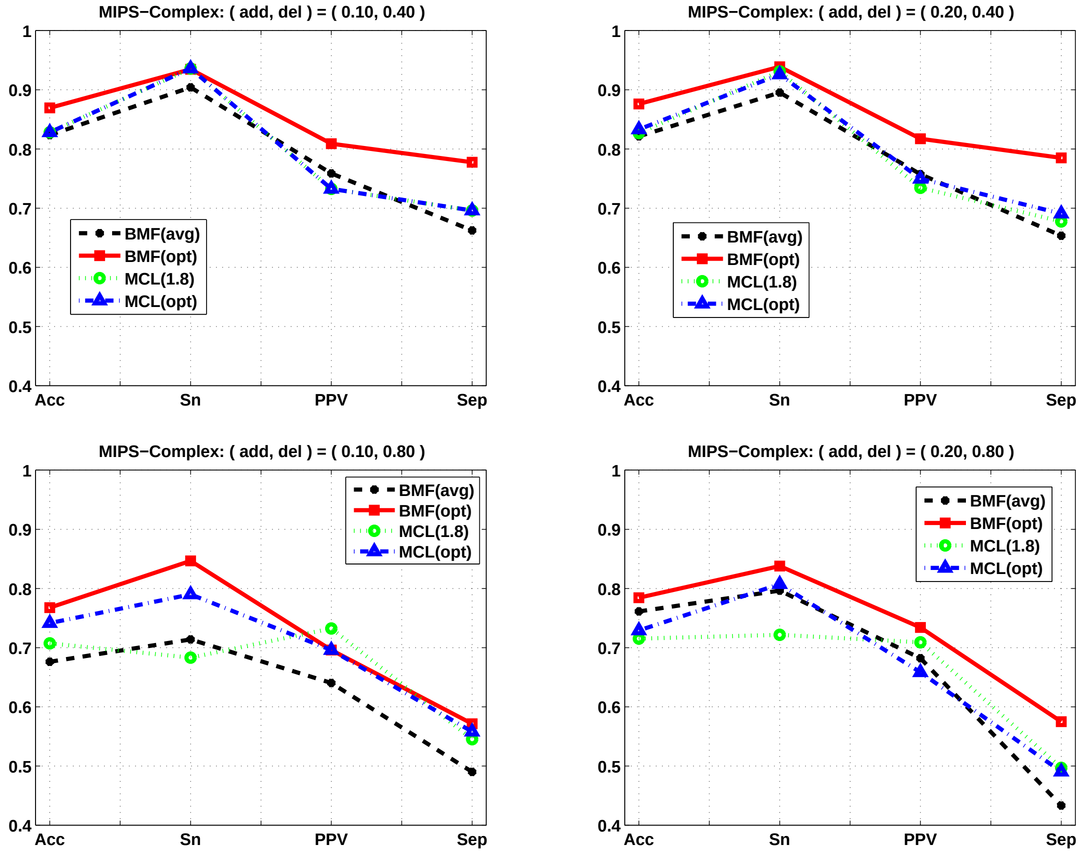

Fig. 6. The evaluations results on 42 percentage pairs (a, d) of random additions and deletions (continue).

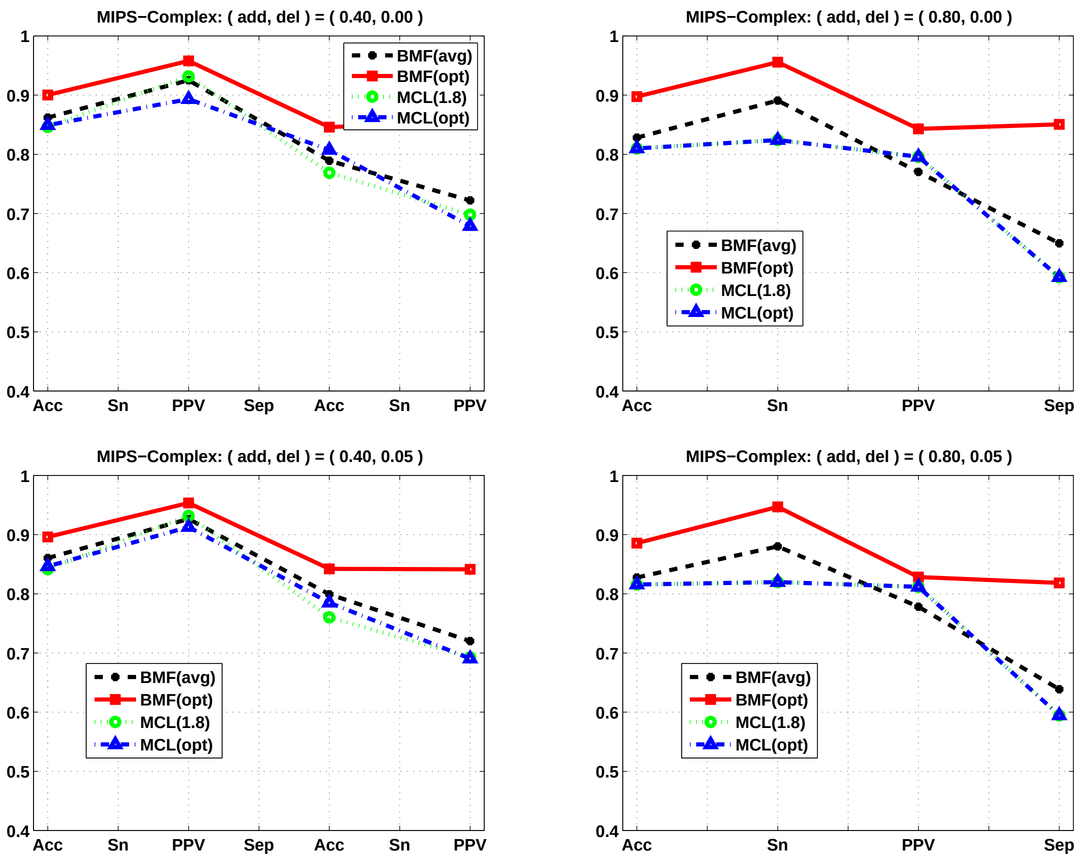

Fig. 7. The evaluations results on 42 percentage pairs (a, d) of random additions and deletions (continue).

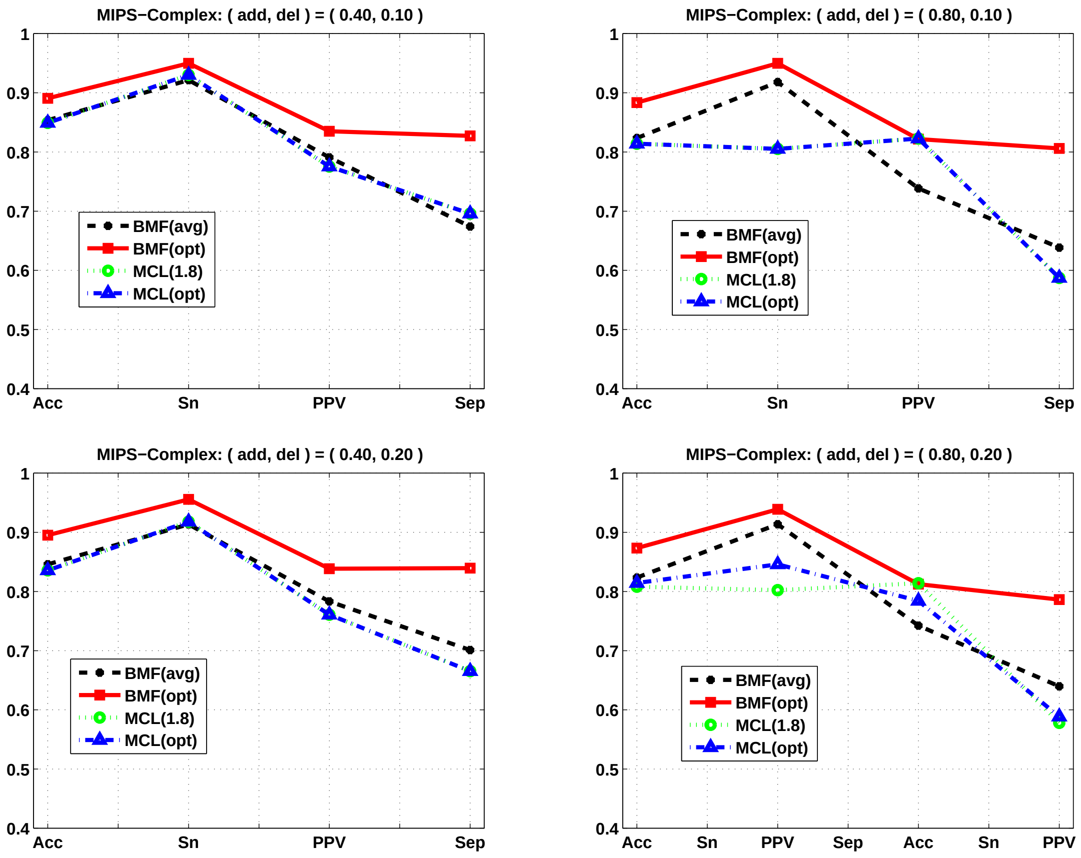

Fig. 8. The evaluations results on 42 percentage pairs (a, d) of random additions and deletions (continue).

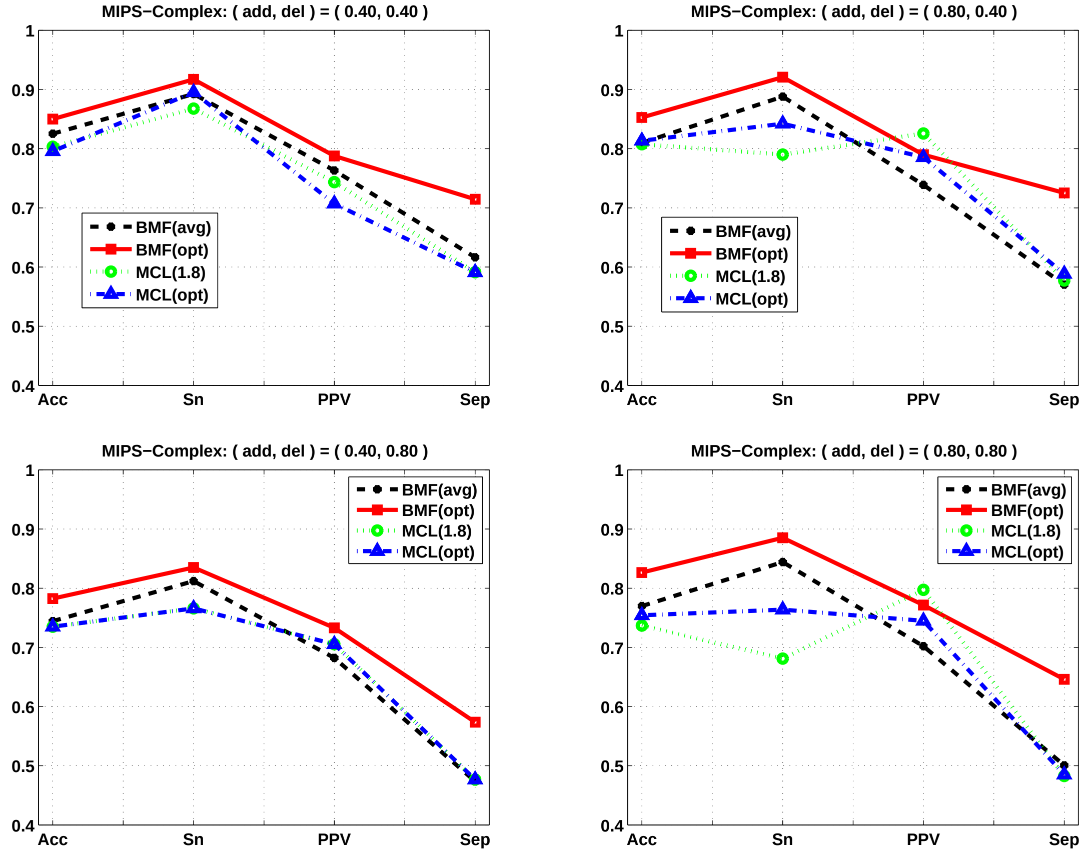

Fig. 9. The evaluations results on 42 percentage pairs (a, d) of random additions and deletions (continue).

which is a discrete optimization over the set $\mathcal{Y}_1 = \{\boldsymbol{y} \in \{0,1\}^m \mid \sum_{j=1}^m y_j = 1\}$ of size m, where for simplicity $\eta = 1$ and $\nu = 0$. Analogously, for estimating $\boldsymbol{A} = [\mathbf{a}_1, \ldots, \mathbf{a}_n]^T$, we have by the following maximization for each $\mathbf{a}_i$:

$$\hat{\mathbf{a}}_i = \arg\max_{\mathbf{a}_i \in \mathcal{A}_1} \ln[q(X|Y, \mathbf{a}_i) q(\mathbf{a}_i|\boldsymbol{\beta})]$$

$$= \arg\max_{\mathbf{a}_i \in \mathcal{A}_1} \left\{ \sum_{t=1}^N [x_{it} \ln(1 - e^{-\mathbf{a}_i^T \boldsymbol{y}_t}) - (1 - x_{it}) \mathbf{a}_i^T \boldsymbol{y}_t] + \mathbf{a}_i^T (\ln \boldsymbol{\beta}) \right\}, \qquad (3)$$

where $\mathcal{A}_1 = \{\mathbf{a} \in \{0,1\}^m \mid \sum_{j=1}^m a_{ij} = 1\}$ is of size m.

$$\boldsymbol{A}^* = \arg\max_{\boldsymbol{A}} \ln \left[\left(\prod_{t=1}^N q(\boldsymbol{x}_t | \boldsymbol{y}_t, \boldsymbol{A}) \right) q(\boldsymbol{A}|\boldsymbol{\beta}) \right]$$

In the "Ying-Step", the maximization for $\boldsymbol{\alpha}$ is made over a simplex, $\sum_{j=1}^m \alpha_j = 1$. Consider the Lagrange function $\mathcal{L}(\boldsymbol{\alpha}, \gamma)$ with a Lagrange multiplier γ,

$$\mathcal{L}(\boldsymbol{\alpha}, \gamma) = \ln \left[\left(\prod_{t=1}^N q(\boldsymbol{y}_t | \boldsymbol{\alpha}) \right) q(\boldsymbol{\alpha}|\Xi) \right] + \gamma(\sum_{j=1}^m \alpha_j - 1),$$

then, it follows from $\frac{\partial \mathcal{L}}{\partial \alpha_j} = 0$ and $\frac{\partial \mathcal{L}}{\partial \gamma} = 0$ that

$$\alpha_j \propto \left(\sum_t y_{jt} \right) + (\xi^\alpha \lambda_j^\alpha - 1) \ln C(\xi^\alpha, \boldsymbol{\lambda}^\alpha), \qquad (4)$$

$$thus, \ \alpha_j = \frac{(\sum_t y_{jt}) + (\xi^\alpha \lambda_j^\alpha - 1) \ln C(\xi^\alpha, \boldsymbol{\lambda}^\alpha)}{\sum_{j=1}^m \left\{ (\sum_t y_{jt}) + (\xi^\alpha \lambda_j^\alpha - 1) \ln C(\xi^\alpha, \boldsymbol{\lambda}^\alpha) \right\}}, \qquad (5)$$

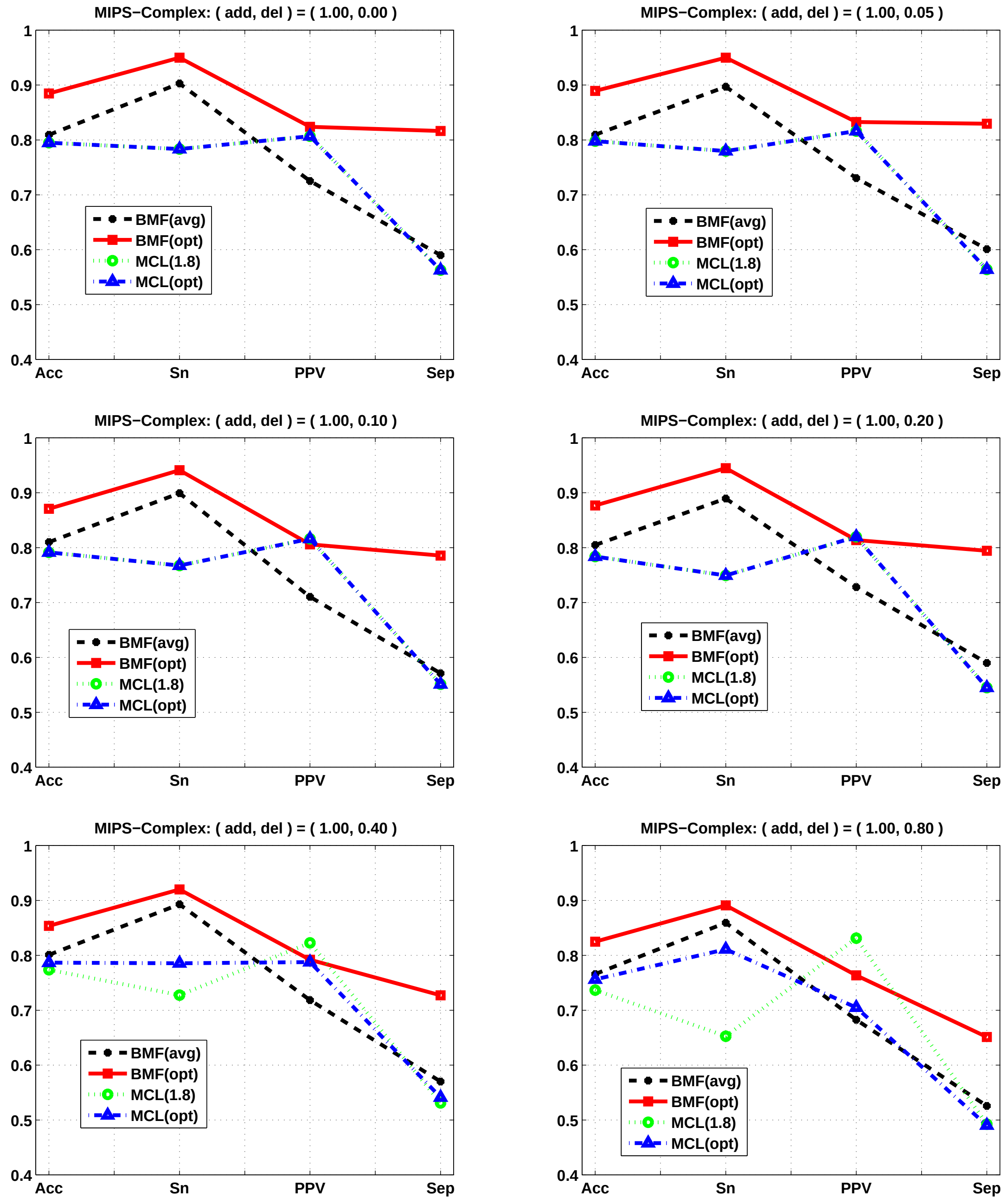

Fig. 10. The evaluations results on 42 percentage pairs (a, d) of random additions and deletions (continue).

where $C(\xi, \boldsymbol{\lambda}) = \Gamma(\xi)/\sum_j \Gamma(\xi\lambda_j)$. Similarly, we get

$$\beta_j = \frac{(\sum_i a_{ij})+(\xi^\beta\lambda_j^\beta-1)\ln C(\xi^\beta,\boldsymbol{\lambda}^\beta)}{\left\{\sum_{j=1}^m(\sum_i a_{ij})+(\xi^\beta\lambda_j^\beta-1)\ln C(\xi^\beta,\boldsymbol{\lambda}^\beta)\right\}}. \tag{6}$$

It can be observed from eq.(5)(6) that the Dirichlet priors produce a regularization.

2.2 A Theoretical Analysis on BYY-BMF

We provide some theoretical results on the efficiency of the algorithm BYY-BMF. Suppose the $n \times N$ binary data matrix X is generated by $X = \boldsymbol{A}^*Y^*$, the underlying low-rank is m^* with $1 \leq m^* < \min\{n, N\}$, and every row of $\boldsymbol{A}^*$ or every column of Y^* takes the value 0 in all dimensions except for one element being 1. For simplicity, we first assume $\boldsymbol{A}^*$ and Y^* are block-diagonal (and so is X), and then show the results still hold when a random permutation is made on X's row or/and column. The m^* blocks of all ones in the block-diagonal X correspond to m non-overlapping biclusters. We denote the sizes of those blocks in order as $n_1 \times N_1, \ldots, n_{m^*} \times N_{m^*}$, where $\sum_{j=1}^{m^*} n_j = n$ and $\sum_{j=1}^{m^*} N_j = N$. Define the following notations: $s_j^r = \sum_{\ell=1}^{j} n_\ell$; $s_j^c = \sum_{\ell=1}^{j} N_\ell$; $\mathcal{B}_j^r = \{i \,|\, s_{j-1}^r + 1 \leq i \leq s_j^r\}$; $\mathcal{B}_j^c = \{t \,|\, s_{j-1}^c + 1 \leq t \leq s_j^c\}$; $s_0^r = s_0^c = 0$.

Theorem 1. *Under the above assumptions with $X = \boldsymbol{A}^*Y^*$, if we initialize $\boldsymbol{A}$ by uniformly randomly assigning 0 or 1 to its entry a_{ij}, initialize $m = m^*$ and $(\forall j)\ \alpha_j = \beta_j = 1/m$ in Algorithm **BYY-BMF**, then **BYY-BMF** converges after only* one *Ying-Yang iteration, and*

$$H(p\|q, \boldsymbol{I}_o) \leq H(p\|q, \boldsymbol{I}_*),\ \forall \boldsymbol{I}_o, \tag{7}$$

*where $H(p\|q, \boldsymbol{I}_o)$ is the harmony measure in eq.(1) calculated by Algorithm **BYY-BMF** from the initialization $\boldsymbol{I}_o = \{\boldsymbol{A}^{(0)}\}$, and $\boldsymbol{I}_* = \{\boldsymbol{A}_*^{(0)}\}$ is an initialization that satisfies eq.(9) and*

$$\{k_j \mid j \in \{1, \ldots, m\}\} = \{1, \ldots, m\}. \tag{8}$$

Moreover, if the "Model-Selection-Step" is used, the resulted $\hat{m}$ from $\boldsymbol{I}_o$ and $\boldsymbol{I}_*$ satisfy $\hat{m}(\boldsymbol{I}_o) \leq \hat{m}(\boldsymbol{I}_*) = m^*$ with equality when eq.(7) is tight.

The result of "one-step-convergence" is based on the above assumption that the data X is generated by $X = \boldsymbol{A}^*Y^*$ with every row of $\boldsymbol{A}^*$ and every column of Y^* taking the value 0 in all elements except for one element being 1. The "one-step-convergence" may not hold without this condition, e.g., when X is corrupted by noise such as $X = \boldsymbol{A}^*Y^* \oplus E$, where $E \in \{0,1\}^{n\times N}$, $\oplus$ is a boolean operator. Based on our experience from experiments, several more steps are usually enough to reach convergence, and the Eq.(7) still holds.

Corollary 1. *Under the Uniform random initialization (as specified in Theorem 1), the probability of the event: "the Algorithm **BYY-BMF** correctly factorize the data matrix X", is approximately $\Pr\{\|X - \hat{\boldsymbol{A}}\hat{Y}\| = 0\} \approx \frac{m!}{m^m}$, where m is initialized at the underlying true number of blocks m^* in X, and $\hat{\boldsymbol{A}}$, $\hat{Y}$ are the output of Algorithm **BYY-BMF**.*

Before proceeding to proofs, we verify the above results in a simulated study first. We use the method described in [3] to generate a synthetic binary data matrix $X_{n\times N}$ with $m^* = 3$ biclusters. The data is considered in three cases: (1) $X1$ (in Fig.11(a)), block-diagonal with non-overlapping biclusters; (2) $X2 = \Pi_r X1 \Pi_c$ (in Fig.11(b)), generated by random permutations Π_r and Π_c on the rows and columns respectively. In the experiments, the *reconstruction error* is evaluated $Err = |X - \hat{X}| = |X - \hat{\boldsymbol{A}}\hat{Y}| = \sum_{i,t} |x_{it} - \hat{\mathbf{a}}_i^T \hat{\boldsymbol{y}}_t|$, where $\hat{\boldsymbol{A}} = [\hat{\mathbf{a}}_1, \ldots, \hat{\mathbf{a}}_n]^T$ and $\hat{Y} = [\hat{\boldsymbol{y}}_1, \ldots, \hat{\boldsymbol{y}}_N]$ are output by Algorithm **BYY-BMF**.

Experimental results are summarized as follows: (1) $X1$ and $X2$ can be reconstructed via $\hat{\boldsymbol{A}}\hat{Y}$ with zero error after only one Ying-Yang iteration if we initialize appropriately with $m_{init} \geq m^*$;

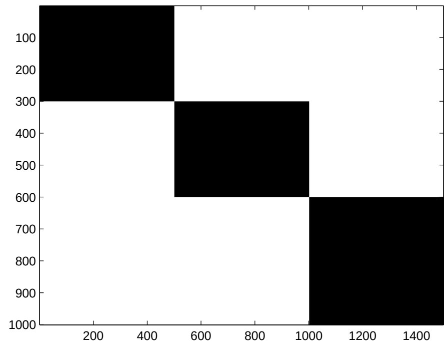

(a) $X1$

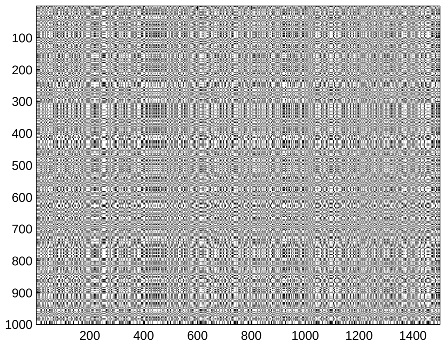

(b) $X2$

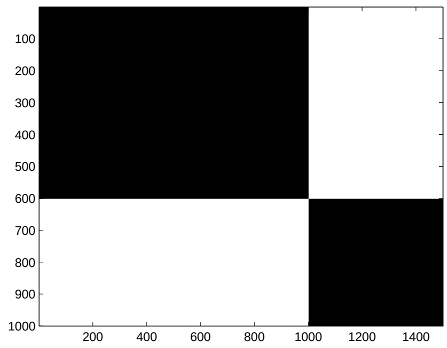

(c) $\widehat{X1}$ by bad initialization

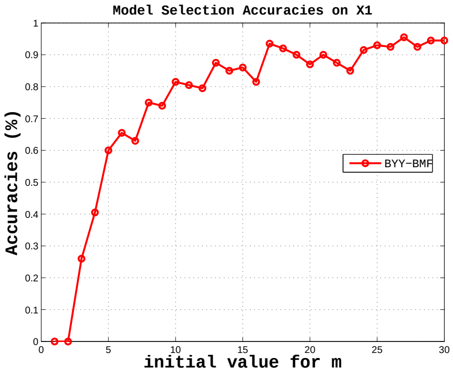

(d) Model selection accuracies on $X1$

Fig. 11. (a) A synthetic data $X_{n\times N}$ with $n = 1000$, $N = 1500$ and $m^* = 3$ underlying biclusters, and "white-black" color corresponding to "$0-1$" entry; (b) $X1$ after a random permutation. Experiments show that the biclusters embedded in the data (a) and (b) can both be correctly recovered. (c) A resulted reconstruction of $X1$ from a bad initialization. (d) Model selection accuracies on $X1$, i.e., $\hat{m} = m^* = 3$, by BYY-BMF with the initial value for m being $1, \ldots, 30$.

(2) m^* can be correctly and automatically detected; (3) Starting from a bad initialization for $X1$ (as indicated in Theorem 1), $m = m_{init} = 3$ will be reduced to $\hat{m} = 2$ as shown in Fig.11(c) and in Fig.12 with a smaller $H(p\|q)$. According to Corollary 1, bad-initialization problem can probably be avoided by selecting the highest $H(p\|q)$ in multiple trials, or by increasing a initial value for m as indicated by the model selection accuracies on $X1$ in Fig.11(d), because a large initial m may probably lead to a set $\mathcal{K} = \{k_j, 1 \le j \le m_{init}\}$ of size $|\mathcal{K}| \ge m^*$, and then the eq.(8) is likely to be satisfied after merging extra k_j according to Lemma 2.

In the following, we prove Theorem 1 by proving several lemmas first.

Lemma 1. *The eq.(2) or eq.(3) can recover the true results if the other quantities are fixed at the true ones. More precisely, fixing $m = m^*$, we have*
(a) If $(\forall j)$ $\alpha_j = 1/m$ and $\boldsymbol{A} = \boldsymbol{A}^*$, then $\hat{Y} = Y^*$, where $\hat{Y} = [\hat{\boldsymbol{y}}_1, \ldots, \hat{\boldsymbol{y}}_N]$ is the estimate by eq.(2).
(b) If $(\forall j)$ $\beta_j = 1/m$ and $Y = Y^*$, then $\hat{\boldsymbol{A}} = \boldsymbol{A}^*$, where $\hat{\boldsymbol{A}} = [\hat{\mathbf{a}}_1, \ldots, \hat{\mathbf{a}}_n]^T$ is the estimate by eq.(3).

Proof: Since $\boldsymbol{A} = \boldsymbol{A}^* = [a_{ij}]_{n\times m}$ is also block-diagonal: $\forall j \in \{1, \ldots, m\}$, $a_{ij} = 1$, if $i \in \mathcal{B}_j^r$; otherwise $a_{ij} = 0$. Enumerate elements in $\mathcal{Y}_1$ in this order: $\boldsymbol{y}^{(1)} = [1, 0, \ldots]^T$, $\boldsymbol{y}^{(2)} = [0, 1, 0, \ldots]^T$, $\ldots$, and then $\boldsymbol{A}\boldsymbol{y}^{(j)} = \boldsymbol{a}_j$, where $\boldsymbol{a}_j = [a_{1j}, \ldots, a_{nj}]^T$ is the j-th column vector of $\boldsymbol{A}$. Then, when $t \in \mathcal{B}_j^c$, the complete log-likelihood of $(\boldsymbol{x}_t, \boldsymbol{y}^{(\ell)})$ is

$$\mathcal{L}(\boldsymbol{x}_t, \boldsymbol{y}^{(\ell)}) = \ln[q(\boldsymbol{x}_t|\boldsymbol{y}^{(\ell)}, \boldsymbol{A})q(\boldsymbol{y}^{(j)}|\boldsymbol{\alpha})] = \begin{cases} n_j \ln(1-e^{-1}) + \ln \alpha_1, & \text{if } \ell = j; \\ n_j \ln(1-e^{0}) - n_\ell + \ln \alpha_\ell, & \text{if } \ell \ne j; \end{cases}$$

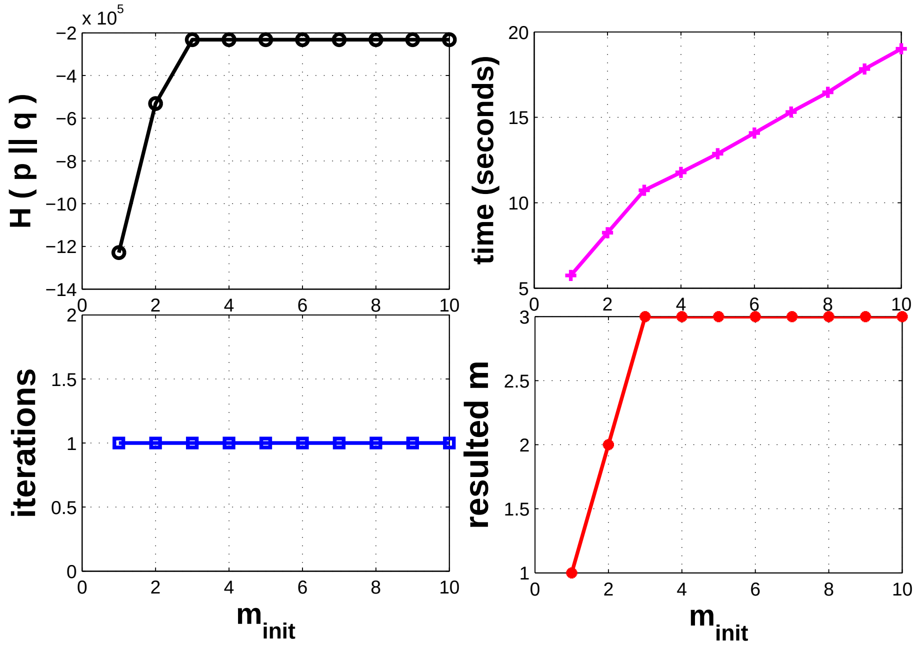

Fig. 12. The horizontal axis is the initial value for the cluster number. The results of **BYY-BMF** on $X1$ coincide with Theorem 1. (All experiments are implemented with Matlab R2006b on Pentium(R) D CPU 3.00GHz, 2.99GHz with 1GB RAM.)

which implies $j = \arg\max_{1\le\ell\le m} \mathcal{L}(\boldsymbol{x}_t, \boldsymbol{y}^{(\ell)})$, or $\hat{\boldsymbol{y}}_t = \boldsymbol{y}^{(j)}$, i.e., $\hat{\boldsymbol{y}}_t$ indicates the correct membership j of $\boldsymbol{x}_t$. Then, we must have $\hat{Y} = Y^*$. The same idea can be used to prove $\hat{A} = \boldsymbol{A}^*$ when $Y = Y^*$. Q.E.D.

Lemma 2. *Initializing $m = m^*$ and $\alpha_j = \beta_j = \frac{1}{m}$, the Algorithm* ***BYY-BMF*** *will converge after only* one *Ying-Yang iteration, due to the following two results:*
(a) If $\boldsymbol{A} = [a_{ij}]_{n\times m}$ satisfies that

$$\forall j \in \{1,\ldots,m\},\ \exists k_j,\ \sum_{i\in\mathcal{B}_j^r} a_{ik_j} > \sum_{i\in\mathcal{B}_j^r} a_{i\ell},\ (\forall \ell) \tag{9}$$

then the resulted $\hat{Y} = [\hat{y}_{jt}]_{m\times N}$ by eq.(2) satisfies

$$\forall j \in \{1,\ldots,m\},\ \hat{y}_{jt} = \begin{cases} 1; \ \textit{if}\ t \in \mathcal{B}_{k_j}^c; \\ 0; \ \textit{otherwise}. \end{cases} \tag{10}$$

(b) If $Y = [y_{jt}]_{m\times N}$ satisfies that

$$\forall j \in \{1,\ldots,m\},\ \exists k_j,\ \sum_{t\in\mathcal{B}_j^c} y_{k_j,t} > \sum_{t\in\mathcal{B}_j^c} y_{\ell t}, (\forall \ell) \tag{11}$$

then the resulted $\hat{\boldsymbol{A}} = [\hat{a}_{ij}]_{n\times m}$ by eq.(3) satisfies

$$\forall j \in \{1,\ldots,m\},\ \hat{a}_{ij} = \begin{cases} 1; \ \textit{if}\ i \in \mathcal{B}_{k_j}^r; \\ 0; \ \textit{otherwise}. \end{cases} \tag{12}$$

Proof: We prove Lemma 2(a) first. Without loss of generality, assume $k_1 = 1$. Then, when $t \in \mathcal{B}_j^c$, the complete log-likelihood of $(\boldsymbol{x}_t, \boldsymbol{y}^\ell)$ is[1]

$$\mathcal{L}(\boldsymbol{x}_t, \boldsymbol{y}^{(\ell)}) = n_{j\ell}^{(1)} \ln(1 - e^{-1}) + n_{j\ell}^{(0)} \ln(1 - e^0) - \tilde{n}_{j\ell}^{(1)} - \tilde{n}_{j\ell}^{(0)} \cdot 0 + \ln(1/m),$$

[1] Here and in the following, we regard $\ln(1 - e^0)$ as $\ln(1 - e^\epsilon)$ with a very small positive value ϵ.

where $n_{j\ell}^{(1)} = \sum_{i\in\mathcal{B}_j^r} a_{i\ell}$, $n_{j\ell}^{(0)} = \sum_{i\in\mathcal{B}_j^r}(1-a_{i\ell})$, and $\tilde{n}_{j\ell}^{(1)} = \sum_{i\notin\mathcal{B}_j^r} a_{i\ell}$, $\tilde{n}_{j\ell}^{(0)} = \sum_{i\notin\mathcal{B}_j^r}(1-a_{i\ell})$, $1\le j,\ell\le m$. The eq.(2) requires to maximize the above log-likelihood with respect to $\ell\in\{1,\dots,m\}$. According to eq.(9), we have

$$k_j = \arg\max_{1\le\ell\le m}\mathcal{L}(\boldsymbol{x}_t, \boldsymbol{y}^{(\ell)}),$$

which implies the eq.(10). This completes the proof for Lemma 2(a). The proof for Lemma 2(b) is similar, and not repeated here.

After initialization $\{\boldsymbol{A}^{(0)}, \alpha_j^{(0)} = \beta_j^{(0)} = 1/m, m = m^*\}$ in Algorithm **BYY-BMF**, if $\boldsymbol{A}^{(0)}$ satisfies eq.(9), then $Y^{(1)}$ is given in eq.(10) by the optimization in Yang-Step. Note that eq.(10) satisfies eq.(11), and thus $\boldsymbol{A}^{(1)}$ is given in eq.(12) by the optimization in Yang-Step. The $\boldsymbol{\alpha}^{(1)}$ and $\boldsymbol{\beta}^{(1)}$ are calculated in Ying-Step. Ignoring the Model-Selection-Step, the Algorithm **BYY-BMF** will produce $Y^{(2)} = Y^{(1)}$, $\boldsymbol{A}^{(2)} = \boldsymbol{A}^{(1)}$ and $\boldsymbol{\alpha}^{(2)} = \boldsymbol{\alpha}^{(1)}$, $\boldsymbol{\beta}^{(2)} = \boldsymbol{\beta}^{(1)}$, i.e., the Algorithm converges. Q.E.D.

Lemma 3. *Permutation properties:*
(a) The solution $X = \boldsymbol{A}Y$ is not unique due to the permutation indeterminacy among the cluster indexes.
*(b) For any permutation matrices Π_r and Π_c respectively on the rows and columns of X, the Algorithm **BYY-BMF** will output $\boldsymbol{A}' = \Pi_r\boldsymbol{A}$ and $Y' = Y\Pi_c$ from the same initialization which produces $X = \boldsymbol{A}Y$.*

Proof: Consider a permutation mapping $\pi : j\in\{1,\dots,m\}\to j'\in\{1,\dots,m\}$, then

$$x_{it} = \mathbf{a}_i^T\boldsymbol{y}_t = \sum_{j=1}^m a_{ij}y_{jt} = \sum_{j=1}^m a_{i,\pi(j)}y_{\pi(j),t} = \sum_{\pi(j)} a_{i,\pi(j)}y_{\pi(j),t} = \sum_{j'} a_{i,j'}y_{j',t},$$

which implies $\boldsymbol{A}_\pi = [a_{ij'}]_{n\times m}$ and $Y_\pi = [y_{j',t}]_{m\times N}$ is also a solution.

Assume the initialization $\boldsymbol{I}^o$ results in $X = \boldsymbol{A}Y$. If we input $X' = \Pi_r X\Pi_c = [x_{i',t'}]_{n\times N}$ into the Algorithm **BYY-BMF**, then starting from $\boldsymbol{I}^o$, all the maximization processes by eq.(2)-(6) are performed on $i'\in\{1,\dots,n\}$ and $t'\in\{1,\dots,N\}$ instead of i and t. The permutation only changes the positions of row elements or column elements, but not change the computation procedure by eq.(2)-(6). Therefore, the output is $\boldsymbol{A}' = [a_{i',j}]_{n\times m} = \Pi_r\boldsymbol{A}$ and $Y' = [y_{jt'}]_{m\times N} = Y\Pi_c$.

Now, we can prove Theorem 1 and Corollary 1 as follows.

Proof of theorem: (Sketched) Without loss of generality, we assume the input data matrix X is block-diagonal, and $\boldsymbol{I}_*$ is such an initialization that $k_j^* = j$, $(\forall j)$. First, we illustrate why eq.(7) holds by a specific initialization $\boldsymbol{I}_o$: $k_j^o = 1$ when $j = 2$; otherwise $k_j^o = j$. Then, the key difference between $H(p\|q, \boldsymbol{I}_o)$ and $H(p\|q, \boldsymbol{I}_*)$ lies in the evaluation on $\{x_{it}\,|\,(i,t)\in\mathcal{D}\}$ with $\mathcal{D} = \{(i,t)\,|\,i\in\mathcal{B}_1^r\wedge t\in\mathcal{B}_2^c;\ i\in\mathcal{B}_2^r\wedge t\in\mathcal{B}_1^c.\}$, i.e.,

$$H(p\|q) = \sum_{(i,t)\in\mathcal{D}}\left\{x_{it}\ln(1-e^{-\hat{\mathbf{a}}_i^T\hat{\boldsymbol{y}}_t}) - (1-x_{it})\hat{\mathbf{a}}_i^T\hat{\boldsymbol{y}}_t\right\} + \dots$$

Then, we have $H(p\|q, \boldsymbol{I}_o) = -(n_1N_2+n_2N_1)\cdot 1+\cdots$ and $H(p\|q, \boldsymbol{I}_*) = -(n_1N_2+n_2N_1)\cdot 0+\cdots$, which implies eq.(7). If "Model-Selection-Step" is used, then it follows from eq.(10) and eq.(12) that: (a) For $\boldsymbol{I}_o$, m will be deducted by at least one because the first two blocks are merged; (b) For $\boldsymbol{I}_*$, m^* blocks are detected.

The complete proof extends the basic idea of this illustration to any initialization $\boldsymbol{I}_o$. We omit the details due to the space limit. Q.E.D.

Proof of corollary: Assume n and N are large, and $n_1\approx\ldots\approx n_m$, $N_1\approx\ldots\approx N_m$. It is reasonable to regard k_j (by eq.(9)) as independently uniformly distributed over $\mathcal{M} = \{1,\dots,m\}$. Then, the number of $\boldsymbol{I}_*$-initializations (which results in correct solutions) is $m!$, while the number of all initializations is m^m. This completes the proof. Q.E.D.

References

1. S. Brohee and J. van Helden, "Evaluation of clustering algorithms for protein-protein interaction networks," *BMC Bioinformatics*, vol. 7, no. 1, p. 488, 2006.
2. ftp://ftpmips.gsf.de/yeast/PPI/PPI 18052006.tab.
3. A. Prelić, S. Bleuler, P. Zimmermann, A. Wille, P. Bühlmann, W. Gruissem, L. Hennig, L. Thiele, and E. Zitzler, "A Systematic Comparison and Evaluation of Biclustering Methods for Gene Expression Data," *Bioinformatics*, vol. 22, no. 9, pp. 1122–1129, 2006.
